# Assessing the challenges to women’s access and implementation of text messages for nutrition behaviour change in rural Tanzania

**DOI:** 10.1017/S1368980020003742

**Published:** 2021-04

**Authors:** Jessica D Rothstein, Rolf Klemm, Debora Niyeha, Erin Smith, Stella Nordhagen

**Affiliations:** 1 Headquarters Nutrition Division, Helen Keller International, Washington, DC, USA; 2 Johns Hopkins Bloomberg School of Public Health, Department of International Health, Social and Behavioral Interventions Program, 615 N. Wolfe St., Room E5038, Baltimore, MD 21205, USA; 3 Tanzania Country Office, Helen Keller International, Dar es Salaam, Tanzania; 4 Global Alliance for Improved Nutrition, Geneva, Switzerland

**Keywords:** Infant and child nutrition, Behaviour, Low-income countries, Nutritional interventions, Qualitative methods, Socio-economic factors

## Abstract

**Objective:**

This process evaluation aimed to understand factors affecting the implementation of a government-sponsored short message service (SMS) programme for delivering nutrition information to rural populations, including message access, acceptability and putting messages into action.

**Design:**

The study was nested within a larger randomised controlled trial. Cross-sectional data collection included structured surveys and in-depth interviews. Data were analysed for key trends and themes using Stata and ATLAS.ti software.

**Setting:**

The study took place in Tanzania’s Mtwara region.

**Participants:**

Surveys were conducted with 205 women and 93 men already enrolled in the randomised controlled trial. A sub-set of 30 women and 14 men participated in the in-depth interviews.

**Results:**

Among women relying on a spouse’s phone, sharing arrangements impeded regular SMS access; men were commonly away from home, forgot to share SMS or did not share them in women’s preferred way. Phone-owning women faced challenges related to charging their phones and defective handsets. Once SMS were delivered, most participants viewed them as trustworthy and comprehensible. However, economic conditions limited the feasibility of applying certain recommendations, such as feeding meat to toddlers. A sub-set of participants concurrently enrolled in an interpersonal counselling (IPC) intervention indicated that the SMS provided reminders of lessons learned during the IPC; yet, the SMS did not help participants contextualise information and overcome the challenges of putting that information into practice.

**Conclusions:**

The challenges to accessing and implementing SMS services highlighted here suggest that such platforms may work well as one component of a comprehensive nutrition intervention, yet not as an isolated effort.

The rapid global adoption of mobile phones has created diverse opportunities to leverage this technology to improve health, referred to as mHealth^(1–3)^. Text messaging, in particular, has considerable potential as an effective channel for behaviour change promotion: it is available on virtually any phone, accessible at any time and can, at relatively low cost, transmit targeted messages^(1,4–6)^. This is particularly true among hard-to-reach or geographically dispersed populations in Sub-Saharan Africa, where mobile phone ownership is growing quickly^(7,8)^.

These factors have driven interest in the use of text messaging to improve maternal, infant and young child nutrition (MIYCN) practices. To date, behaviour change interventions targeting optimal prenatal nutrition, breast-feeding and complementary feeding practices have primarily relied on individual counselling, group-based education and mass media strategies^(9–16)^. Text messaging interventions may be particularly well suited to support key MIYCN behaviours because messages can be easily tailored to the target parents’ circumstances (e.g. stage of pregnancy) or the child’s age^(17)^. Short message service (SMS) text messages may be employed as a stand-alone intervention or as part of a broader strategy that integrates targeted SMS with two-way communication and/or interpersonal activities.

Small studies evaluating the efficacy of SMS on nutrition behaviours have shown improved knowledge of infant feeding practices^(18,19)^ and improved breast-feeding behaviours^(20–23)^, but many of these studies have been conducted in controlled settings, with intensive resources over a short duration. There is a need to understand how SMS programmes operate in real-world settings, including whether and how participants receive, accept and understand messages.

Figure 1 illustrates the flow of information in such programmes, from SMS generation to effects on behaviour. Several environmental, economic and socio-cultural barriers may interfere with the intended flow of information into action. First, the timely and appropriate delivery of SMS (step 1 in Fig. 1) that are generated by mobile network operators may be impeded by inconsistent network connectivity or technical challenges related to the mHealth platform itself^(24)^. For example, an evaluation of a maternal health SMS programme in South Africa showed that only about 80 % of expected messages were received by registered users^(25)^.

**Fig. 1:**
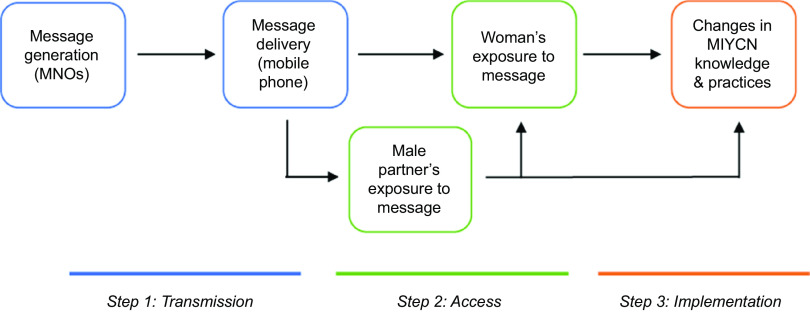
Information flow of client-directed text messages for nutrition behaviour change

Once an SMS has been successfully delivered to a mobile phone, another set of barriers may affect target clients’ exposure to the message (step 2 in Fig. 1). Common challenges in rural and resource-poor settings include limited access to electricity, which may cause phones to be turned off for extended periods of time, and difficulties affording enough phone credit to maintain service^(24,26)^. Access to SMS may be further complicated by low rates of phone ownership among women, especially in rural areas, and a recent analysis based on Demographic and Health Survey data revealed that female mobile phone ownership is <50 % in several sub-Saharan African countries^(27)^. In Tanzania, where nearly two-thirds of the population lives in rural areas, slightly more than half of all women own a phone, and the mobile ‘gender gap’ is 17 %^(27,28)^. In response, a number of mHealth interventions focusing on child health and nutrition have enrolled and targeted male partners, yet challenges to accessing SMS among women persist^(25,29,30)^. Previous qualitative studies have shed light on the intersection of gender dynamics and phone sharing; in rural Uganda, for example, male phone owners often refused to let a woman use the phone independently due to concerns about her accessing their personal information within it^(31,32)^.

Finally, several factors potentially influence the extent to which SMS are translated into knowledge and behaviour change (step 3 in Fig. 1). Overly technical language or poor credibility may threaten messages’ acceptability, which is essential for their implementation^(25,33)^. A minimum number of messages may be required for there to be an effect on behaviour, or, conversely, over-saturation and ‘recipient fatigue’ may discourage clients from following the recommended practices^(26,34)^.

Little research exists on how such factors may impact the effectiveness of mobile nutrition interventions. Closer consideration of clients’ perspectives on and engagement with nutrition-related SMS is essential for understanding the barriers to their real-world implementation and for optimising their future design. Here we aim to fill this gap by reporting findings from a process evaluation focused on understanding clients’ receipt, comprehension, acceptability and perceptions of the SMS, as well as their ability to put the MIYCN recommendations into practice.

## Materials and methods

### Study design

**Fig. 2:**
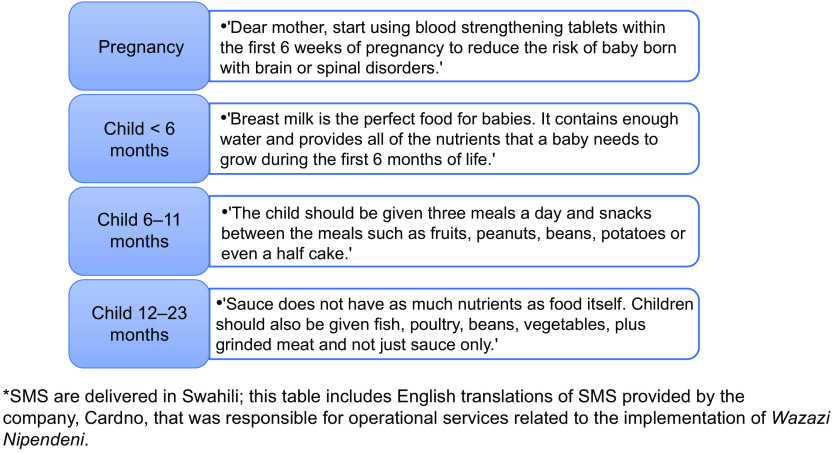
Examples of *Wazazi Nipendeni* short message service (SMS) by life stage (translated from Swahili to English)

The study was implemented within a larger randomised controlled trial (RCT) that aims to evaluate the effectiveness and cost-effectiveness of the Tanzanian Government’s *Wazazi Nipendeni* programme, with and without interpersonal counselling (IPC), on nutrition knowledge, practices and outcomes^(35)^. From February through April 2018, pregnant women and mothers of children <12 months, along with their male partners, were randomised to one of four study arms: the *Wazazi Nipendeni* SMS service, (‘SMS arm’), individual and group counselling and education using the Tanzanian government’s *Mkoba wa Siku 1000* curriculum (‘IPC arm’), a combination of both SMS and IPC interventions (‘SMS + IPC arm’) or usual care (i.e. neither intervention). For participants in the SMS and SMS + IPC arms, data collectors registered their mobile phones and/or a phone that they had access to with the *Wazazi Nipendeni* service, which is free to subscribers. The service provides SMS with health information and reminders that are timed to arrive when they may influence practices, from pregnancy until the child’s fifth birthday, with some key messages sent repeatedly. Message frequency declines with the age of the child. Examples of *Wazazi Nipendeni* SMS are provided in Fig. 2 and in Supplemental Table 1 (see online supplementary material).

The present study was conducted in April 2019, approximately 12 months after the interventions began, in order to understand the access, receptivity and reactions of study enrollees to the messages. The experiences of participants receiving the SMS service alongside the IPC intervention, including perceived similarities and differences between the two intervention modalities, were also explored. We used both quantitative (survey) and qualitative (in-depth interview; IDI) methods to objectively assess these topics while also exploring individual experiences. The study was carried out in Mtwara region – a rural, historically underserved region in Tanzania’s southern corner where the agricultural economy centres around the annual cashew harvest. Regional indicators of malnutrition are slightly worse than national averages: stunting affects 37·7 % of children <5 years in Mtwara, as compared with 34·4 % country wide. Children and women of reproductive age suffer from higher-than-average rates of anaemia (58·6 % and 47·1 %, respectively)^(28)^.

### Participants and sample selection

Study participants were a subsample of the approximately 1200 women and 700 men enrolled in the SMS and SMS + IPC arms of the RCT. Women were eligible for inclusion in the process evaluation if they owned or had access to a phone that had been registered with *Wazazi Nipendeni* during enrollment into the RCT and had received at least one SMS from the service (either by reading it herself or by having someone share it with her) since enrollment. Likewise, men were eligible to participate if they were enrolled in the RCT and owned a registered phone that had received at least one *Wazazi Nipendeni* SMS. Exclusion criteria for all participants were the inability to provide informed consent or unwillingness to participate in the process evaluation. In addition, individuals < 16 years of age had been excluded from enrollment in the RCT.

The target sample size for the survey was estimated to be 300 individuals, including 200 women and 100 men. This sample size was deemed sufficient to measure key proportions with a precision of ±10 percentage points and is similar in size to that used by other mHealth surveys^(20,36)^. Furthermore, of the 200 women, we aimed to recruit approximately 100 women who owned their own mobile phone enrolled in *Wazazi Nipendeni* and 100 women who did not own a phone but had access to a household member’s phone enrolled in *Wazazi Nipendeni*; the inclusion of these two groups allowed us to explore specific experiences related to phone ownership as compared with reliance on another person’s phone. The target sample size for the IDI was approximately forty-five participants (thirty women, fifteen men); this sample size was deemed sufficient to reach thematic saturation based on criteria outlined by Malterud *et al.*^(37)^, including relatively specific aims and strong dialogue between interviewers and interviewees. Given that the RCT’s study population exhibited minimal ethnic and religious diversity, a larger sample size was not deemed necessary.

Our sample selection consisted of several steps. First, five villages each from the SMS and SMS + IPC arms of the RCT (ten villages total) were selected through random number generation. Next, 150 potential participants (100 women, 50 men) were randomly selected from each of the SMS and SMS + IPC arms based on eligibility criteria (i.e. enrollment in *Wazazi Nipendeni* and receipt of SMS) and targeted traits (i.e. study arm membership and phone ownership). Participants’ phone ownership status was determined from the RCT’s baseline questionnaire data. Finally, a total of forty-five participants (thirty women, fifteen men) from the SMS and SMS + IPC arms were randomly selected from the list of survey participants to participate in the IDI in addition to the survey. The selection of IDI participants was based on the same targeted traits used in selecting survey participants to ensure that a variety of perspectives would be represented in the qualitative data.

To recruit participants, field supervisors conducted home visits with the assistance of local leaders using information on the participant’s hamlet and name. After verifying an individual’s eligibility, field supervisors provided a brief explanation of the goals and procedures of the process evaluation. Eligible and interested individuals were subsequently visited at home by a data collector who administered the survey directly after obtaining written informed consent. If a selected participant was not home or unavailable during the initial visit, a maximum of two follow-up attempts were made. In cases where a selected participant was ineligible or not reachable after three attempts, he or she was replaced by a randomly selected replacement.

Data collectors were provided with information on whether a survey participant had been randomly selected for participation in the IDI prior to visiting the household. For those individuals, the data collector first administered the survey and then asked the participant whether he/she was interested in participating in a longer interview. In practice, data collectors were asked to use some discretion in choosing IDI participants, such that a randomly selected individual who was particularly reticent to speak or rushed during the survey was not ultimately invited to participate in the IDI; this procedure was used to ensure rich qualitative data. In those cases, replacement IDI participants were randomly selected.

### Data collection

Surveys and IDI were conducted in Swahili by a team of eight local data collectors who had obtained a minimum of a bachelor’s degree and had prior experience working in Mtwara. The team was rigorously trained in both quantitative and qualitative methods to ensure standardised data collection, with pre-testing to ensure the appropriateness and clarity of all questions. Data collection activities took place in a private location in or around participants’ homes. In line with locally appropriate practices, participants were compensated with a small snack as a token of appreciation for their time and collaboration; the compensation did not differ for those participating in the IDI. Throughout data collection, two field supervisors conducted quality control by visiting a subsample of households and re-administering specific survey questions to ensure reliability of the information collected. In addition, they continuously monitored interview activities to ensure that appropriate interviewing techniques (e.g. probing, non-verbal behaviour, active listening) were used and that bias was not introduced. Supervisors provided individualised feedback as needed, and the research manager led debriefing meetings with the team following each day of data collection.

#### Cross-sectional survey

Survey data were recorded using tablets. Questions were close-ended and assessed participants’ access and exposure to the SMS; perceptions of the tone, clarity and other aspects of message content; and experiences acting on the recommendations. The survey instrument for men included additional questions about sharing messages with their wives. For women, the instrument included separate modules for phone-owning and non-phone-owning women (the latter group had a partner’s or other household member’s phone registered with *Wazazi Nipendeni*). Both instruments included a separate module for participants enrolled in the SMS + IPC arm and a section in which the interviewer asked the participant to open his/her phone and display prior messages to determine whether messages had been received and read. Socio-demographic characteristics were captured upon enrollment into the RCT, including age, education, employment, marital status, household composition and household infrastructure and assets.

#### In-depth interviews

The IDI addressed similar topics as the survey while encouraging participants to provide narrative accounts and explanations for their preferences and opinions. Each IDI took place immediately after the survey and was usually conducted by the same data collector. Prior to initiating the interview, the data collector introduced it as an opportunity for participants to speak freely about their thoughts and reactions to *Wazazi Nipendeni* and explained that there were no right or wrong answers.

IDI were steered by a semi-structured interview guide, consisting of open-ended questions and follow-up probes to elicit descriptions and storytelling (see online supplementary material, Supplemental Table 2). For several questions, a participant’s survey responses informed the selection of follow-up topics. Several IDI questions invited participants to openly recall-specific SMS, such as those that were preferred and not preferred, those that they could act on *v*. those that they were unable to act on and those that proved useful. We anticipated that these detailed examples from participants’ personal experiences would provide context for interpreting the survey data while deepening our understanding of the topics of interest. During IDI, the data collector also sent pre-selected example *Wazazi Nipendeni* SMS to participants’ phones or to an example phone and asked the participant to summarise the SMS and discuss the steps that they would take to put it into practice. This interactive exercise was used to provide insight into the factors influencing how *Wazazi Nipendeni* SMS are interpreted and implemented.

IDI were audio-recorded and documented through interviewers’ field notes; the recordings lasted on average 29 min (range 19–48 min). Directly following each IDI, data collectors were required to write expanded interview notes to document observations not captured elsewhere, including the interview environment, impressions of the participant, any issues that emerged and key take-aways from the participant’s responses.

### Data analysis

Cross-sectional survey data were analysed using Stata 13 (StataCorp. LP). Distributions of variables were characterised by frequency or by mean and sd and disaggregated by sex and, where relevant, by phone ownership status or study arm. Wealth quintiles were calculated with the Equity Tool, which uses ten parameters related to household infrastructure and assets based on the 2015 Tanzania Demographic and Health Survey^(28,38)^. Food insecurity was estimated using the Household Food Insecurity Access Scale^(39)^ and analysed as a categorical variable. Significance of statistical associations of sex, phone ownership status, study arm and wealth quintile with other variables of interest was analysed via *χ*^2^ tests.

Native Swahili speakers transcribed the IDI recordings verbatim and simultaneously translated them into English. The transcripts were supplemented with interviewers’ field notes and coded by the first author, a trained and experienced qualitative researcher, using ATLAS.ti software (Scientific Software Development). An initial coding framework was developed consisting of a priori codes drawn from the IDI guides and inductive codes for themes and relationships surfacing from the text. This led to the development of a codebook including code definitions, guidelines for their application and example text segments. The first author then returned to each transcript to consistently apply the final codes. Following procedures for directed content analysis described by Hsieh & Shannon^(40)^, codes were organised into categories, sub-themes and broader themes linking the underlying relationships among categories. The first author used memo writing to intensify engagement with the material and examine the data for patterns during this process. Feedback was elicited from the team of data collectors through participatory meetings at several stages to confirm sound interpretations of the data, enhancing the credibility and confirmability of the analysis^(41)^.

### Ethical approval

Written informed consent (including, where relevant, for audio recording) was obtained from all study participants prior to enrollment in the present study. Participants were identified by anonymous ID numbers, and data confidentiality was ensured at all levels.

## Results

We triangulated relevant quantitative and qualitative findings to ascertain the defining features of participants’ perceptions and experiences with *Wazazi Nipendeni*. Below, we begin with an overview of participant characteristics and then present findings thematically, drawing on illustrative direct quotations from participants when appropriate.

### Participant characteristics

A total of 205 women and 93 men participated in the study. Basic demographic information is displayed in Table 1. All male participants owned phones, while 92 (44·9 %) female participants who did not own a phone had access to a household member’s phone. Most respondents were literate and had completed primary school; however, many households were poor or food insecure. There were no significant differences among participants in the two study arms (data not shown), aside from educational attainment among women: a greater percentage of women in the SMS arm had completed secondary school as compared with those in the SMS + IPC arms (15·2 % and 4·4 %, respectively; *P* = 0·009; data not shown).

**Table 1:** Socio-demographic characteristics and receipt of *Wazazi Nipendeni* short message service (SMS) among study participants (*n* 298)*

	Women (*n* 205)		Men (*n* 93)	
Variable	*n*	%	*n*	%
Characteristics of participants				
Age in years				
Mean	27·6		31·8
sd	7·5		8·1
Mobile phone ownership				
Owns phone	98	47·8	93	100·0
Owns phone and has access to additional phone(s)	15	7·3	0	0
Does not own phone but has access to phone(s)	92	44·9	0	0
Education				
None/primary school incomplete	36	17·6	9	9·7
Primary school complete	148	72·2	67	72·0
Secondary school complete or higher	19	9·3	16	17·3
Missing	2	1·0	1	1·1
Swahili literacy				
Cannot read	8	6·5	4	4·3
Can read	116	93·6	89	95·7
RCT study arm				
SMS arm	92	44·9	39	41·9
SMS + IPC arm	113	55·1	54	58·1
Marital status of women				
Husband/partner lives with her	183	89·7	–	
Husband/partner lives elsewhere	7	3·4		
Not married or cohabiting	14	6·9		
Household infrastructure and socio-economic status				
Number of household members				
Mean	4·8		4·5	
sd	1·8		1·7	
Number of children in household				
Mean	2·3		2·1	
sd	1·4		1·2	
Source of drinking water				
Dug well without pump	73	35·8	43	46·2
Rainwater	98	48·0	40	43·0
Other improved source	20	9·8	7	7·5
Other unimproved source	13	6·4	3	3·2
Improved sanitation facility	32	15·7	13	14·0
Electricity in household	180	88·3	85	91·4
Household in lowest two quintiles of wealth*	52	25·4	17	18·3
Household food insecurity access category				
Food secure	102	49·8	48	51·6
Mildly food insecure	13	6·3	5	5·4
Moderately food insecure	55	26·8	30	32·3
Severely food insecure	35	17·1	10	10·8
Receipt of *Wazazi Nipendeni* text messages				
Number of WN messages received over the past 30 d				
Zero	83	40·5	38	40·9
1 or 2 messages	18	8·8	10	10·8
Between 3 and 10 messages	56	27·3	24	25·8
More than 10 messages	26	12·7	14	15·1
Don’t know/don’t remember	22	10·7	7	7·5
Number of WN messages received over the past week				
Zero	100	48·8	46	49·5
1 message	33	16·1	11	11·8
2–3 messages	26	12·7	23	24·7
More than 3 messages	24	11·7	9	9·7
Don’t know/don’t remember	22	10·7	4	4·3
Time elapsed after WN registration before first message was received				
Within 1–2 d	54	26·3	14	15·1
Within 1 week	66	32·2	38	40·9
After 1 week, within 2 months	64	31·2	29	31·2
After 3 or more months	6	2·9	2	2·2
Don’t know/don’t remember	15	7·3	10	10·8

RCT, randomised controlled trial; IPC, interpersonal counselling; WN, *Wazazi Nipendeni*.

* National wealth quintiles were calculated using the Equity Tool from the 2015 Tanzania DHS.

The socio-demographic characteristics of the thirty women and fourteen men who participated in the IDI reflected those of the general study population, except that female IDI participants were slightly older and less educated (26·7 % did not complete primary school) than women in the larger sample (data not shown).

All of those surveyed had, as planned, been exposed to *Wazazi Nipendeni*, albeit not always regularly. The majority of male and female study participants reported receiving their first message from *Wazazi Nipendeni* within 1 week of being registered. Nearly half of participants reported that they had not received any messages from the service over the past 7 d, while 28·8 % of women and 36·5 % of men received one to three messages during that time.

Overall response rates for the survey were 71·7 % and 51·1 % for women and men, respectively. Of the original 200 women selected from the RCT study population, 163 (81 %) met the eligibility criteria (i.e. had received at least one SMS from *Wazazi Nipendeni)*, and 113 (69·3 %) of those were recruited. An additional 123 (80·6 %) women were eligible from 155 randomly selected replacement women, and 92 (74·8 %) were recruited. Of the original 100 selected men, 86 (86·0 %) were eligible, and 42 (48·8 %) of those men were recruited; after visiting 117 randomly selected replacements, of which 96 (82·1 %) were eligible, an additional 51 (53·1 %) men were recruited.

### Access and exposure to text messages

#### Challenges related to phone charging and defective handsets

According to female study participants who owned their own phone, maintaining fully charged batteries was the most significant challenge to regularly accessing *Wazazi Nipendeni* SMS. Of 113 phone-owning women surveyed, 24 (21·2 %) charged their phones away from home, with most leaving their phones for several hours (up to two full days) to charge (see online supplementary material, Supplemental Table 3). For women who charged their phones at home, widespread use of solar energy meant unreliable electricity, particularly during the rainy season. As one respondent explained,

*Sometimes [the phone] is without charge for even three days. When there is not enough sunlight, the battery cannot get fully charged…it all depends on the dispersion of clouds and getting sunshine.*

(30 years old, primary school complete, 14-month-old child)

In her opinion, she had missed recent SMS from *Wazazi Nipendeni* due to her phone being off. Over half of female survey participants reported that their phone shuts off multiple times each month because of insufficient charge.

In addition, issues related to handsets interfered with some phone-owning women’s message access. About a quarter (23·9 %) mentioned low-quality batteries as an issue, and several IDI participants reported that phone batteries held charge for less than a day. Defective keypads and screens were reported by smaller numbers of women (see online supplementary material, Supplemental Table 3), with phone repairs often prohibitively expensive.

#### Challenges related to lack of phone ownership

For women who did not own a phone, difficulties accessing information from *Wazazi Nipendeni* were more pronounced. While most non-phone-owning women reported access to their husband’s or male partner’s phone, substantial variability was observed in message sharing: while nearly half of these women reported that messages were shared with them several times per week, 19·8 % stated they had only been exposed to the messages on one or two occasions (Table 2).

**Table 2:** Access to short message service (SMS) among non-phone-owning women (*N* 81)

Variable	*n*	%
Owner of phone to which woman has access		
Husband/male partner	76	93·8
Female household member	3	3·7
Neighbour	2	2·5
Woman’s ability to use phone		
Can use phone without asking permission	18	22·2
Must ask permission to use phone	63	77·8
Frequency with which woman uses phone		
Never	13	16·1
Rarely (once/month or less)	19	23·5
Occasionally (2–4 times/month)	6	7·4
A few times per week	13	16·1
Every day	30	37·0
Frequency with which phone owner shares SMS with woman		
Rarely (maximum of 1–2 times in total)	16	19·8
About once/month	8	9·9
Several times/month	7	8·6
About once/week	12	14·8
Several times/week	37	45·7
Missing	1	1·2
How phone owner most commonly shares SMS with woman		
Reads SMS aloud	30	37·0
Shows phone directly to her	36	44·4
Paraphrases/summarises SMS	12	14·8
Missing	3	3·7
Woman is shown WN-SMS in her preferred way	48	59·3

WN, *Wazazi Nipendeni*.

Since most non-phone-owning women (77·8 %) could not use the phone without permission, their SMS access was subject to the phone owner’s discretion and availability. In some cases, limited message sharing was likely a consequence of men’s limited engagement with the messages themselves. As displayed in Table 3, 11·9 % of men reported never or only occasionally opening the SMS, 15·1 % reported never or occasionally reading them and 57·0 % reported never or occasionally keeping them in order to re-read later. Men were significantly less likely than phone-owning women to read the SMS and to keep them to re-read later (*P* = 0·010 and *P* = 0·048, respectively).

**Table 3:** Comparison of exposure to short message service (SMS) after delivery among phone-owning women and men

	Phone-owning women (*n* 113)		Men (*n* 93)		
	*n*	%	*n*	%	*P*
Open the SMS					
Never	0		1	1·1	0·069
Occasionally	3	2·7	10	10·8	
Often	11	9·7	7	7·5	
Always	99	87·6	75	80·7	
Read the SMS					
Never	1	0·9	6	6·5	0·010
Occasionally	2	1·8	8	8·6	
Often	13	11·5	6	6·5	
Always	97	85·8	73	78·5	
Keep the SMS to read it again later					
Never	33	29·2	42	45·2	0·048
Occasionally	27	23·89	11	11·8	
Often	15	13·3	10	10·8	
Always	38	33·6	30	32·3	

In other cases, non-phone-owning women’s access to SMS was restricted by their partners’ economic and social activities. In the IDI, several women mentioned that their partners often left early for the farm, travelled or socialised elsewhere, while women spent most of the day at home. As one interviewee explained,

*You know, using someone else’s property is not convenient. This is my husband, but he is busy! That is why he ends up giving me the messages in pieces. But if it were my own phone, I would find my own time to read them freely.*

(39 years old, primary school complete, 9-month-old child)

Among male survey participants who reported ‘never’ or ‘occasionally’ sharing SMS with their wife (*n* 35), the most commonly cited reasons were not being home when the message was delivered and forgetting to share it. Only a small number of men (*n* 2) reported not understanding the SMS themselves or believing that their spouse would not benefit from the SMS as the motive for not sharing them.

Even when messages were shared, women were not always able to receive and process the nutrition recommendations as they desired. When asked how the phone owner most commonly shared SMS, most women reported being read the message aloud or being shown the phone; paraphrasing was less common (14·8 %). Contrasting this information with women’s responses to a question on their preferences for how messages should be shared revealed substantial discordance: more than 40 % of non-phone-owning women were not able to access the SMS in their preferred way (Table 2). For example, of the fifty-one non-phone-owning women who preferred to read the message from the phone, only thirty were given this opportunity.

### Perceptions of message tone, clarity and message content

#### Trust and comprehension of SMS

Participants almost universally agreed that the SMS were user-friendly, relevant, easy to understand and trustworthy (Table 4). During the IDI, participants often referred to the SMS sender as ‘experts’ and recounted specific lessons and skills they were learning. Women and men alike most frequently recalled *Wazazi Nipendeni* guidelines surrounding exclusive breast-feeding, handwashing and preparing nutritious porridge. Several participants compared these recommendations to their previous knowledge and commented on changes they had observed in their children, which, in turn, motivated them to continue reading the SMS.

*There was a message suggesting me to give my child a heavy porridge mixed with things like groundnuts. Previously … I was just raising (my older son) using my own understanding; for instance, I used to give him a soft porridge, it had nothing in it except salt or sugar. Things have changed now. You can notice a difference between my two children, this one and the other one…. I know this child will have better health.*

(32 years old, primary school complete, 18-month-old child)

**Table 4:** Perceptions of short message service (SMS) among women and men

	Women (*n* 205)		Men (*n* 93)		
Variable	*n*	%	*n*	%	*P*
Perceives SMS as easy to understand	198	96·6	90	96·8	0·933
Enjoys reading the SMS overall	195	95·1	88	94·6	0·716
Perceives SMS as relevant to their life	200	97·6	93	100·0	0·129
Finds the tone of the SMS to be friendly	201	98·1	89	95·7	0·245
Feels as though they can trust the SMS to be accurate	200	97·6	92	98·9	0·437
Finds the recommendation(s) in the SMS to be confusing	46	22·4	17	18·3	0·415
Perceives that some of the practices that the SMS recommend would be impossible to do	107	52·2	62	66·7	0·019
Perceives the SMS to be too long	62	30·2	19	20·4	0·078
Believes there should be fewer SMS or they should be sent less frequently	28	13·7	28	30·1	0·001
Feels as though it is more important for her husband to get the SMS than for her to get them	41	20·0	–		–

Interviewees noted several SMS features that enhanced their comprehension and trust. First, it was easy to recognise that the SMS were sent from a corporate number, not a personal one. Second, the messages’ life-stage-specific tailoring helped simplify feeding guidelines, as one mother explained that this made it clear ‘*what and how to feed a baby at what time*.’ Finally, nearly all interviewees expressed appreciation for the repetition of certain SMS up to three or four times, which underscored their importance and made them easier to remember.

*It is very helpful because there are people who have difficulties understanding something instantly. Such people need to be reminded by sending a message more than once.*

(30 years old, primary school complete, 14-month-old child)

While the perceptions of the SMS themselves were largely positive, some participants appeared dissatisfied by the number and frequency of the messages. These sentiments were more common among male participants: as displayed in Table 4, 30·1 % of men and 13·7 % of women believed that there should be fewer SMS or they should be sent less frequently (*P* = 0·001).

#### Confusion surrounding SMS recommendations

Despite the SMS service’s overall acceptability, close to one-fifth of female and male survey participants reported being confused by an SMS recommendation in the past (Table 4). Qualitative data suggested that a main source of this confusion was the use of unfamiliar words: during the exercise in which the data collector shared example SMS with participants and asked them to explain the messages, for example, a number of participants pointed out that they did not understand the Swahili word for ‘snacks’ and did not know what ‘yams’ were.

### Implementing the nutrition recommendations

#### Economic challenges

Successful implementation of many messages was hindered by households’ economic conditions. Over half of women and two-thirds of men agreed that some of the messages’ recommendations were impossible to carry out, with the differences between the genders being significant (*P* = 0·019) (Table 4). The principle barrier was the limited accessibility of certain recommended foods. Most commonly, IDI participants discussed the challenges of incorporating animal products into children’s diets. Women and men alike recalled SMS that explained such foods’ nutrient value and encouraged parents to add items such as minced meat, liver, milk or eggs to children’s meals. Yet given that animal rearing was rare in the study communities, meat had to be purchased; it was therefore unattainable for families facing financial constraints. One single mother lamented,

*I liked the lessons. We always like our kids to be healthy, but the challenge has been having money to spend… We don’t have milk. Meat—we can buy this once money is available, but we don’t have it right now. If I had the money, I could have worked on all of them [the SMS recommendations] for my child.*

(31 years old, primary school incomplete, 17-month-old child)

#### Limited availability of foods

SMS recommendations referring to nutrient-rich produce items also proved difficult to implement. According to interviewees, many fruits and vegetables were seasonal, while others were only available in different ecological zones. This created uncertainty, as a 25-year-old mother explained, ‘*I don’t understand when they advise us the kind of foods to give a baby though such foods are not available where I live.’*

#### Consequences of issues with implementing recommendations

Participants’ inability to implement many food-related recommendations, due to limited availability or affordability, led to feelings of helplessness and unease. Several IDI participants expressed concerns for their children’s health. One man explained,

*If I miss out on those food products, it makes me think that my child will not score well health-wise because he didn’t get the goods in time.… I want to get a balanced diet for my child. How do I do that if I don’t have financial capability?*

(25 years old, secondary school complete, 23-month-old child)

As many recommendations called for decisions surrounding money (i.e. buying nutrient-dense foods), women often did not feel comfortable as the only targeted client. ‘*I am not a businesswoman*,’ one woman insisted, ‘*I depend on someone else*.’ As displayed in Table 4, 20·2 % of women felt it was more important for their husbands to receive the SMS than them. Among some phone-owning women, these circumstances motivated them to share the SMS with their husbands, as one 33-year-old mother explained, *‘Perhaps after receiving those messages (my husband) might see their importance and find something to give the child.’*

### Relationship between SMS and interpersonal counselling intervention

#### Alignment of intervention content

The experiences of study participants enrolled in the SMS + IPC arm of the RCT shed light on how concurrent participation in the interpersonal intervention affected participants’ perceptions of the SMS service and the similarities and differences between the two modalities. Engagement with the IPC activities, which involved group meetings led by community health workers (CHW) and individual home visits, was high among target women: 112 of 113 (99·1 %) surveyed women from the SMS + IPC arm reported attending past meetings. Among men, attendance was lower, with only twenty-three (42·6 %) of fifty-four surveyed men from the SMS + IPC arm having participated.

All SMS + IPC female participants agreed that the SMS content reflected the CHW’ lessons, and 95·5 % stated that they did not find it confusing to be receiving information from multiple sources (see online supplementary material, Supplemental Table 4). Interviewees regularly discussed how recommendations surrounding breast-feeding, meal and snack frequency, dietary diversity and hygiene from the two sources aligned. Some participants even believed the two types of information came from the same source:

*After getting those* [Wazazi Nipendeni] *messages; I can also go to* [Mkoba wa Siku] *meetings, and you see the kind of discussions they have are all more or less the same. The teacher who instructs in this side [pointing to phone] is the same with the one who comes to me. There’s no difference. You know they are equally intelligent.*

(38 years old, primary school incomplete, 21-month-old child)

In this context, messages often reinforced and reminded participants of lessons taught by CHW. Most SMS + IPC women stated that messages had reminded them to do something they learned about in IPC meetings on at least four occasions, with one-quarter of these women recalling that this had occurred more than ten times (see online supplementary material, Supplemental Table 4).

#### Advantages of the interpersonal counselling model

Despite the similarities, participants discussed several advantages of the IPC activities compared with SMS. CHW offered more detailed recommendations and utilised learning tools, such as radios and pictures, to communicate in appealing ways. In addition, CHW could provide more thorough explanations and respond to individualised questions during home visits. Several other IDI participants recalled that the CHW would sometimes observe participants preparing food and feeding their children to ensure that they were able to adhere to the suggestions.

When challenges arose, CHW could offer counselling and problem-solving strategies. For example, one woman recounted that when she had trouble breast-feeding her child, the CHW showed her the correct positioning and technique. Furthermore, the group setting facilitated open discussion of problems related to obtaining suggested foods and strategies for overcoming them.

*When we meet in groups, we are free; we learn from each other and solve the challenges that arise. If you don’t get one [food], you have another. If you don’t have meat, you get sardines—they are all important!*

(27 years old, primary school complete, 13-month-old child)

## Discussion

In this study, we aimed to understand the challenges to accessing and implementing an SMS service to improve MIYCN behaviours in rural Tanzania by examining broad trends as well as individual experiences. Findings suggest that the feasibility of regularly accessing text messages is hindered by communities’ socio-economic realities. Among phone-owning women, engagement with *Wazazi Nipendeni* SMS was frequently complicated by charging needs, defective mobile handsets and maintenance problems. These findings align with those of other studies from sub-Saharan Africa and South Asia documenting the abundance of low-quality or secondhand devices among women and the lack of enabling infrastructure^(24,42,43)^.

Women who relied on a male partner’s phone had to navigate another set of barriers to accessing SMS since they were generally prohibited from using the phone on their own. According to the non-phone-owning women in our study, nearly 20 % of spouses had shared the *Wazazi Nipendeni* SMS with them only one or two times at most. These findings are consistent with other studies, including an unrelated qualitative evaluation of the implementation of *Wazazi Nipendeni* in Tanzania’s Iringa region, which found that husbands rarely shared messages with their wives^(44)^. In rural India, Hazra and colleagues^(45)^ indicated that only 34 % of men who registered for a voice messaging intervention actually listened to the messages, and less than half of those men shared the information with their wives. Our study participants indicated that men’s work and social activities outside of the home were the main barriers to women’s message exposure. Such experiences suggest that the descriptors of phone ‘ownership’ and ‘access’ are not as clear-cut as they may seem.

In order to achieve their fullest potential, services such as *Wazazi Nipendeni* should explore strategies for improving male partners’ engagement and rates of message sharing. Several interventions, for example, have aimed to align the timing of message delivery with male enrollees’ schedules, either through assessing preferences prior to implementation or by incorporating a feature into the registration process allowing clients to select specific days and times for message delivery^(46–48)^. Another strategy for improving men’s participation involves targeting them with specific messages that encourage the discussion of nutrition recommendations with their wives^(45)^. Male engagement with the SMS content may also be heightened by integrating additional topics that are of particular interest to men^(49)^. Research conducted in conjunction with the RCT suggested that men would be more likely to attend the CHW-led IPC meetings if topics such as farming and money management were discussed; future research should explore whether this type of approach would be effective in the context of mobile interventions. Given that men are often key decision-makers in matters related to children’s health and nutrition and, as phone-owners, serve as gatekeepers to SMS content, their active engagement is critical to mHealth programmes’ success^(50–52)^.

Our process evaluation also revealed challenges related to participants’ capacity to put nutrition recommendations into action following the successful receipt of *Wazazi Nipendeni* SMS. This appeared to vary based on the costs associated with specific behaviours. Overall, interviewees did not voice any barriers to recommended practices that required little or no financial investment, such as exclusive breast-feeding or mixing ground peanuts into children’s porridge. In contrast, practices that required purchasing animal products or finding fruits and vegetables that were not locally grown proved impossible for many. For parents who were learning the importance of feeding their children diverse and protein-rich diets for the first time, the lack of actionable steps led to feelings of frustration, helplessness and guilt; such reactions threaten to discourage clients from engaging with the service.

Experiences like these point to the limitations of employing a text messaging programme as an isolated intervention. Although study participants found the *Wazazi Nipendeni* SMS highly acceptable and credible, when confusions emerged, the messages could not provide clarifications or suggest adaptations. In contrast, study participants enrolled in the SMS + IPC arm of the RCT believed that the group discussions and home visits helped participants to contextualise the nutrition advice and, ultimately, enabled behaviour change. Our qualitative data highlight multiple features of interpersonal interactions that cannot be replaced by a one-way text messaging service, such as hands-on demonstrations, responding to questions and encouraging clients to overcome initial challenges.

These study findings suggest several ways that the SMS service could be optimised to more appropriately meet communities’ needs. Messages that mention specific foods should include affordable and locally available, seasonally appropriate alternatives as well as guidance and examples of substitutions. Small-scale formative research studies play a crucial role in ensuring that intervention content aligns with clients’ geographic and socio-economic contexts^(46)^. In Senegal, for example, Downs and colleagues^(47)^ conducted a series of focus group discussions to understand local food production and seasonal variation during the design of an mHealth voice messaging intervention to improve child feeding practices. SMS content could also be tailored to certain agricultural regions or seasons, just as they are currently tailored to users’ life stage based on information requested during registration; this may allow for larger-scale programmes to be effectively contextualised to local or regional levels. Refining message content in these ways has the potential to empower parents to provide their children with higher-quality diets even amid economic constraints.

In addition, enhancing the interactive nature of *Wazazi Nipendeni* and similar SMS programmes would likely increase clients’ engagement. Considering that study participants valued the demonstrations and discussions that characterised IPC activities, strategies for connecting mobile phone users with health workers should be explored. Previously, interventions have incorporated telephone communication through either health worker-initiated calls or through the availability of a toll-free hotline; several studies of such approaches have indicated high rates of user satisfaction and positive behavioural outcomes^(22,29,53,54)^. Two-way messaging through a virtual helpdesk offers another mechanism through which mHealth clients may interactively seek information. In Kenya, Unger and colleagues^(48)^ compared a one-way SMS to a two-way SMS intervention for breast-feeding promotion and found that mothers enrolled in the two-way channel were significantly more likely to sustain exclusive breast-feeding for the child’s first 6 months of life. This effect was attributed to how the two-way channel assisted women in overcoming social pressures and other breast-feeding challenges. There is also evidence that maintenance of two-way messaging is achievable on a larger scale: MomConnect, a nationally scaled mHealth intervention in South Africa focused on maternal and child health, offers a helpdesk feature managed by a registered nurse where users may ask questions or provide feedback on healthcare services^(55)^. A recent evaluation found that although it was not widely advertised, approximately 8 % of over 95 000 MomConnect users utilised the helpdesk, with the average response time being <24 h^(56)^. Future studies should explore the feasibility of building these additional functionalities into the *Wazazi Nipendeni* platform and their effect on perceived message actionability.

Ultimately, however, our study participant’s experiences suggest that a chief barrier to improving MIYCN practices – the prohibitive costs of meat and other nutrient-rich foods – is outside the scope of an SMS programme to address. It may be necessary for interventions to go beyond the provision of information, reminders and even two-way channels and provide inputs in support of the purchase and consumption of recommended foods. Interventions that have integrated behaviour change communication with monetary or nonmonetary incentives have demonstrated a positive impact on practices related to child health^(57–59)^. The growth of phone ownership in low- and middle-income countries, mobile applications and mobile network operators have integrated financial services, referred to as mobile money, which may be leveraged to distribute monetary or airtime incentives^(34,60)^. In one RCT, Gibson and colleagues^(61)^ demonstrated that coupling SMS reminders with mobile cash transfers significantly improved Kenyan infants’ vaccination rates as compared with a control group. The potential for integrating mobile-money incentives into SMS programmes focused on nutrition should be explored. In addition, it may be instructive to join mHealth services with other types of social assistance, such as microcredit or job training programmes, to increase their impact. In Nigeria, for example, the integration of weekly SMS and voice messages into microcredit groups resulted in greater adherence to recommended breast-feeding practices^(21)^. By creating a more enabling environment for nutrition behaviour change, integrated models such as these may alleviate the barriers to acceptance and implementation of SMS recommendations experienced by our study participants.

This study faced several limitations. First, we did not examine factors affecting SMS delivery (step 1 on the pathway in Fig. 1) due to a lack of data available on whether *Wazazi Nipendeni* messages sent by the mobile network operators actually arrived at clients’ phones. However, 18·5 % of the women and 14·0 % of the men who were randomly selected for this study were ineligible due to never having received an SMS from *Wazazi Nipendeni* – despite having ostensibly been enrolled in it during the baseline of the RCT. This, along with findings from other evaluations in sub-Saharan Africa^(62,63)^, suggest that technical obstacles to message delivery are worth examining further. Second, our data were collected cross-sectionally via self-report and therefore may have been subject to recall and/or social desirability bias. However, the use of mixed methods allowed us to triangulate key findings, improving the reliability of our conclusions^(64)^. Third, while the exclusion of potentially reticent participants helped to improve data richness, it may have excluded the views of some, if reticence to respond to an interview is correlated with level of engagement with the SMS intervention. Finally, the study was conducted in only ten villages from Mtwara and therefore may not be generalisable outside of this region. Nevertheless, the socio-economic conditions, nutrition-related needs and mobile phone ownership rate among our study population are similar to those in other parts of rural East Africa; thus, our findings are likely relevant to the development and diffusion of mHealth programmes in other settings.

Despite these limitations, this study provides valuable insights into multiple barriers to achieving the full impact of SMS-based behaviour change communication on MIYCN practices in rural Tanzania. Ultimately, our findings suggest that one-way text messaging interventions are not equipped to replace face-to-face nutrition advice. However, they may be well suited to be one component of a comprehensive intervention strategy, providing reminders and reinforcements for lessons learned in other contexts, connecting clients to interactive helpdesks or hotlines and/or complementing other social assistance efforts. Participants’ widespread agreement that the *Wazazi Nipendeni* content closely reflects the IPC curriculum suggests potential for a formalised, scaled-up integration of these two government-sponsored programmes, should the eventual results of the RCT impact evaluation demonstrate that their combination proved effective. The future research agenda surrounding mHealth for MIYCN should also explore strategies for tailoring message content to local needs, encouraging greater male engagement and creating interactive platforms to foster positive nutrition behaviours.

## Supplementary material

For supplementary material accompanying this paper visit https://doi.org/10.1017/S1368980020003742. click here to view supplementary material
