# Supplementary material for: Assessing the challenges to women’s access and implementation of text messages for nutrition behaviour change in rural Tanzania
Source: Public Health Nutr. 2020 Oct 29;24(6):1478–91. doi: 10.1017/S1368980020003742 (PMC8025099; doi:10.1017/S1368980020003742)
Supplement: Supplementary file 1 [file S1368980020003742sup001.docx]

*Supplement 1. Example SMS sent to* Wazazi Nipendeni *enrollees*

| **Targeted life stage** | **SMS*** |
| --- | --- |
| Pregnancy | Dear mother, start using blood strengthening tablets within the first 6 weeks of pregnancy to reduce the risk of baby born with brain or spinal disorders. |
|  | It is important to eat a variety of foods each day. Try to eat foods like animal products, vegetables, fruits, cereals, roots and bananas for a nutritious diet. |
|  | Dear mother, make sure you eat 3 meals per day and snacks between the 3 meals. Eating a variety of food strengthens your health and helps in fetal development. |
|  | It is very important to start breastfeeding within 1 hour after delivery. The baby should be exposed to the early yellow milk to prevent the disease. |
| When child is <6 mo. of age | Dear mother, remember to breastfeed your baby as she needs, day and night. This helps to have plenty of breast milk for the first 6 months. |
|  | Breast milk is the perfect food for babies. It contains enough water and provides all of the nutrients that a baby needs to grow during the first 6 months of life. |
|  | The fact is that breastfeeding mothers are among of the most nutritious demanding individuals. Food allocation should be prioritized to them. |
|  | Hello mama, how is your baby? Do you feel that baby is not getting enough milk? Consult a health worker if you have problems with breastfeeding. |
| When child is 6-11 mo. | The child should be given three meals a day and snacks between the meals such as fruits, peanuts, beans, potatoes or even a half cake. |
|  | Mother, feed your child with a mixture of corn, meat, peanut butter, vegetables and fruits. |
|  | Even after the baby is 6 months old, mother's milk continues to give her baby protection against various diseases such as diarrhea, asthma diseases, etc. |
|  | Remember to wash hands with clean water and soap before preparing a meal, after toilet and before feeding the baby to prevent the baby from contaminated diseases. |
| When child is 12-23 mo. | Sauce does not have as much nutrients as food itself. Children should also be given fish, poultry, beans, vegetables, plus grinded meat and not just sauce only. |
|  | Cooking and food storage equipment for the baby can be a source of contamination and diseases. Make sure they are washed with fresh water and soap before using it. |
|  | Education and advice on good care for children are provided at the clinic. The little baby's clinic gives you the knowledge and skills to improve your child's nutrition and health. |
|  | Dear mother, keep feeding your baby during night and day until it reaches 2 or more years. Your baby still gets plenty of nutrients from your milk. |

*SMS are delivered in Kiswahili; this table includes English translations of SMS provided by the company, Cardno, that was responsible for operational services related to the implementation of *Wazazi Nipendeni*.

*Supplement 2. In-depth Interview Guide for Female Participants*

*ENUMERATOR READ: During this interview I am hoping to learn more about your own experiences with the WN service. The questions that I’m going to ask do not have right or wrong answers. We are simply interested in learning about your experiences, opinions, and thoughts. The interview will last about 30-45 minutes.*

*ENUMERATOR INSTRUCTIONS: After asking each primary question, use the follow-up questions (bullet points) to gather additional details. Questions marked as “OPTIONAL” may be used at your own discretion as needed.*

**Part A. Perceptions of messages (all women)**

*ENUMERATOR READ: To begin, I’m interested in hearing a bit more about how you understand the SMS from WN.*

A_1. *If participant noted not reading SMS regularly in survey:* I noticed in the survey that you often do not read the WN text messages. Could you explain to me in more detail why?

- Is there anything that would make you more likely to read them or would make it easier for you to do so?

A_2. Which SMS, if any, do you remember?

- Which SMS, if any, have you found to be the most useful?
- Which SMS, if any, have you liked the most? Why?
- Which SMS, if any, have you liked the least? Why?

A_3. Overall, have you been able to understand the advice and recommendations provided in the SMS?

- Is the wording in the SMS clear?
- Do the messages seem relevant to you?
- Can you think of an example of a message you couldn’t understand or didn’t think was clear? If you have it on your phone, could you show it to me?

A_4. Would you say that it has been easy or challenging to put the recommended nutrition behaviors into practice, or somewhere in between? Why?

- Are the suggestions generally feasible, given the realities of your life?
- Are the types of foods or supplements mentioned in the messages accessible from where you live?
  - *If respondent cannot remember any specific SMS about types of foods, probe by giving her the following examples:*

|  | **English SMS** | **Swahili SMS** |
| --- | --- | --- |
| 1 | "Mother feed your child with a mixture of corn, meat, peanut butter, vegetables and fruits." | Mama mlishe mtoto chakula mchanganyiko chenye nafaka, nyama, jamii ya kunde,mboga  mboga na matunda. |
| 2 | Animal originated food stuff has a lot of nutrients. Dear mother, remember to include beef, fish, poultry, eggs, milk or seafood to baby foods | Vyakula vya asili ya wanyama vina virutubishi vingi. Mpendwa mama, kumbuka kuongeza nyama, samaki, kuku, mayai, maziwa au dagaa kwenye vyakula vya mtoto |

- Are the types of services mentioned in the messages available at your local dispensary/health facility?
  - *If respondent cannot remember any specific SMS about types of services, probe by giving her the following examples:*

|  | **English SMS** | **Swahili SMS** |
| --- | --- | --- |
| 1 | Make sure you have you been given iron and folic acid pills. You should take these every day for the entire duration of your pregnancy to keep you healthy and ensure your baby is developing well. | Mama hakikisha umepewa vidonge vya kuongeza damu. Vitumie kila siku kipindi chote cha ujauzito kwa afya yako na maendeleo ya mtoto aliye tumboni |
| 2 | Dear parent / guardian, don’t worry. Anti tapeworm medication are available free of charge at health care centers. Do not miss your child this important service! | Mpendwa mzazi/mlezi, usihofu. Dawa za kutibu minyoo hupatikana bila malipo kwenye vituo vinavyotoa huduma za afya. Usimkoseshe mtoto wako huduma hii muhimu! |

A_5. Can you think of an SMS that you have received from WN that you were able to act on (i.e., put into practice the recommendation)?

- What was the recommendation about?
- What did you do as a result?
  - Did you do that just once, or did you keep doing it?
  - For how long?
- What helped you to follow through?
- What made it difficult to do so?
- Do you think that making that change/acting that way had a positive or negative effect on yourself or your child? Please explain.

A_6. Can you think of an SMS that you have received from WN that you were *not* able to translate into practice?

- What was the recommendation about?
- What were the barriers to putting the recommendation into practice?

A_7. When you are not able to put a recommendation into practice, how does that make you feel?

- Does this affect your interest in reading SMS from WN in the future? If so, how?

A_8. Some people enrolled in WN believe that they receive too many messages, while others feel like they don’t receive enough messages and would like to receive them more frequently. Would you prefer to receive more or less SMS from WN? Why?

- Do you ever get the same message more than once? If so, what did you think of that?
- Would it be helpful to receive a given message more than once? Why / why not?

A_9. In order to get a better understanding of how women react when they receive an SMS from WN, I’m going to ask you to guide me through the process of reading an SMS and thinking through the next steps to take. *If woman owns a phone:* To do this, I’m going to send a “pretend” SMS to your mobile phone. *If woman does not own a phone and is literate:* To do this, I’m going to show you a “pretend” SMS on a phone I have. *If woman does not own a phone and is illiterate:* To do this, I’m going to read a “pretend” SMS aloud to you word for word.

- How would you summarize the SMS? What is it recommending? What could you do as a result?
- Is there anything in the message that is confusing?
- Do you already practice this recommendation?
- What would your next step be in order to put this recommendation into practice? And next?
- Are there any barriers to putting it into practice?
- What do you think would be the benefits of putting it into practice?
- What might be the drawbacks of putting it into practice?
- Do you think you would succeed in putting this message into practice? Why / why not?

A_10. Overall, do you trust the information that comes from WN? Why / why not?

- *If no*: What would help you to trust the WN service more?
- Have there been any particular messages that you did not believe or did not trust? If so, please explain and provide an example if possible.
- Did any messages contradict something you already knew or had heard elsewhere? If so, please explain and provide an example if possible.

A_11. *If participant noted sharing WN messages/content in survey:* I noticed in the survey that you sometimes share the messages or their content with other people. Could you explain that to me in a bit more detail?

- With whom do you share the messages?
- How do you show him/her/them the messages?
- About how often do you do this?
- When do you do this?
- What is the reason for sharing the messages?
- How do the other people normally react when you share the information?
  - Have you ever gotten a negative reaction from them?

**Part B. Access to text messages (phone-owning women)**

*ENUMERATOR READ: Now I’m going to ask a few questions related to how you are able to access SMS on your own phone.*

B_1. In the survey, we asked about any problems that you have with your mobile phone handset, such as a cracked screen, a defective key pad, or poor-quality batteries. If you have experienced any of these problems, could you tell me a little more about them?

- Has the phone always had this issue?
  - If not, for how long has this been a problem?
- *OPTIONAL*: How does this issue affect how you use your phone?
- *ENUMERATOR: ask to see the issue on the phone and note its severity*

B_2. If you have had any other problems related to your handset that we have not mentioned yet, please describe those.

- Do you think any of these might have influenced whether you got the WN messages or how well you could read them? If so, please explain.

B_3. Do you think it would be useful for another person in your household to get messages, *in addition to* you? Why / why not?

B_4. Do you think it would be useful for another person in your household to get messages, *instead of* you? Why / why not?

**Part C. Access to text messages (non-phone-owning women)**

*ENUMERATOR READ: Now I’m going to ask some questions to learn more about how the owner of the phone that you have access to shares SMS with you.*

C_1. Thinking back to the last time that your male partner/other household member (person that owns the phone that is registered with WN) shared an SMS from WN with you, how did he/she do it? Please describe the whole interaction in terms of how he/she notified you that the SMS had been delivered, how he/she showed, read, or summarized the information, and whether you discussed it afterwards.

- Was this reflective of how he/she usually shares SMS with you?
  - If not, how is it normally done?
- Would you prefer for this person to share the SMS from WN in a different way? How?

C_2. Do you think it is useful for this person to get messages, instead of or in addition to you? Why / why not?

- Is there anyone else in your household or community who you would like to get these messages?
  - Who?
  - Why?

C_3. In your opinion, is your access to SMS from WN limited by the phone owner’s personal concerns or work demands? Please describe.

- *OPTIONAL:* Barriers could include concerns such as the phone owner not trusting you or having to split time between different co-wives, or the phone owner’s work demands such as being at the farm all day.

**Part D. Relationship between SMS and IPC content (women in SMS+IPC arm)**

*ENUMERATOR READ [if participant reported attending MwS sessions or being visited at home by a CHW in survey]: I’m also interested in the experiences that you have had with the Mkoba Wa Siku program with the community health worker (CHW) in your village.*

D_1. Please describe your experiences with the Mkoba wa Siku sessions and/or the CHW’s home visits.

- *OPTIONAL*: Have you enjoyed the sessions and/or the home visits? Why / why not?
- What is your favorite part of the sessions and/or the home visits?
- What is your least favorite part of the sessions and/or the home visits?
- Have you been able to understand the advice provided during the sessions and/or the home visits? Was there anything you didn’t understand?

D_2. Have you made any changes in your life as a result of things you have learned from the CHW? If so, please explain.

- Have you noticed any changes in your child as a result of things you have learned from the CHW? If so, please explain.

D_3. Has the CHW ever mentioned the WN messages?

- Have you ever asked him/her about something in them?
- Has anyone else in the group session done this?

D_4. Thinking about what you have learned from the CHW and the information provided in the SMS from WN, have the recommendations for health and nutrition practices been generally the same across the two sources, or different? Or a mix of similar and different? Please explain.

- Can you think of any examples of recommendations provided by the SMS that were not provided by the CHW? Or vice versa?
- Were there any WN messages that seemed to contradict what you’d heard from the CHW? Or vice versa?
- Overall, do the SMS remind you of things that you have already learned about from the CHW, or do they provide new recommendations that you haven’t heard about?

D_5. Is it easier to understand and follow advice provided by the CHW during the MwS sessions, or that provided in the SMS from WN? Please explain.

D_6. What would you think of men attending the MwS sessions? Why?

*Supplement 3. Issues affecting access to SMS among phone-owning women (N=113)*

| **Variable** | **N(%)** |
| --- | --- |
| Location of phone charging  At home  Away from home | 89 (78.8)  24 (21.2) |
| Length of time that woman must leave phone at the place away from home to charge it (N=23)  A few hours  More than a few hours, less than a full day  1-2 full days | 5 (21.7)  16 (69.6)  2 (8.7) |

| Frequency of phone shutting off because it does not have enough charge (N=112)  Once/month or less frequently  More than once/month | 53 (47.3)  59 (52.7) |
| --- | --- |
| Issues related to mobile handset that make it difficult to use  Screen  Keypad  Battery quality | 14 (12.4)  16 (14.2)  27 (23.9) |

*Supplement 4. Perspectives on the relationship between* Wazazi Nipendeni *SMS and Mkoba wa Siku 1000 sessions among women in SMS+IPC arm*

| **Variable** | **N (%)** |
| --- | --- |
| ***Level of agreement with the following statements (N=111)*** | |
| “Overall, the SMS reflect what is taught by the CHW”  Strongly agree  Somewhat agree  Somewhat disagree  Strongly disagree | 105 (94.6)  6 (5.4)  0  0 |
| “Learning about similar topics from the SMS and the MwS sessions has given me more trust in the messages”  Strongly agree  Somewhat agree  Somewhat disagree  Strongly disagree | 103 (92.8)  8 (7.2)  0  0 |
| “It is confusing to be receiving information on nutrition and health from those two sources”  Strongly agree  Somewhat agree  Somewhat disagree  Strongly disagree | 3 (2.7)  2 (1.8)  10 (9.0)  96 (86.5) |
| ***Frequency of the following experiences (N=112)*** | |
| SMS served as a reminder to do something that woman first learned about in the MwS sessions  Never  Once or twice  Several times (4-10 times)  Many times (more than 10 times) | 10 (9.0)  34 (30.6)  39 (35.1)  28 (25.2) |
| Woman received an SMS that has contradicted what was taught in the MwS sessions  Never  Once or twice  Several times (4-10 times)  Many times (more than 10 times)  Missing | 107 (95.4)  2 (1.8)  0  2 (1.8)  1 (0.9) |
| Woman discussed the SMS during an MwS session  Never  Once or twice  Several times (4-10 times)  Many times (more than 10 times)  Missing | 65 (58.0)  21 (18.8)  15 (13.4)  10 (8.9)  1 (0.9) |
| Woman asked the CHW about something as a result of an SMS  Never  Once or twice  Several times (4-10 times)  Many times (more than 10 times) | 86 (76.8)  20 (17.9)  4 (3.6)  2 (1.8) |
